# Glad Greetings

**Published:** 1875-01

**Authors:** 


*Glad Greetings*

Of the New Year to one and all! As the sands of the hour-glass of Time counted out the last hours of the twelve months, that are gone and buried forever in the dark crypts of the Past, how many thousand hearts, pulsating within the great, throbbing bosom of our humanity, looked back with varied emotions upon the scenes of the Old Year, that have passed before us like a kaleidoscopic vision! How many bright hopes have fled—how many tears have fallen upon the graves of loved ones, now sleeping the dreamless slumber of the dead! And how many worthy ambitions have been crushed by the relentless grasp of Poverty, or the cold selfish-of those who heed not merit unless it be surrounded by the glitter of wealth!

The usual amount of crime and murder has desolated homes and broken hearts; rapine has defiantly committed its lawless deeds of violence; demagogism has sought to ride astride the shoulders of Liberty to the throne of its own sordid and selfish aggrandizement; and red-handed War, ever grasping and insatiate as its compatriot, Death, has, in many parts of the world, brought its usual miserable train of anarchy, desolation, poverty, and weeping widows and orphans.

But, thanks be to the kind and supreme Ruler of the Universe, behind the dark curtain of the Old Year there flashes many a fair picture of beauty and joy, and there is scarcely one heart but what can, upon retrospection, find in the days that are now no more, some precious memento, some glad and golden hours to treasure among the memories of the "beautiful past."

Though death, in the last year, has claimed men distinguished in science, literature, or the annals of statesmanship; and though stars of effulgent beauty and brightness in the world of art and music have set to rise no more on this lower sphere; yet others as gifted will take their places, and add to the irresistible impetus of the grand and ever onward and upward march of progress through the densely-peopled ranks of Christian civilization. Science, with which there is never any retrograde movement, has achieved victories in the past year whose beneficial influence will be felt by the present and all successive generations. Religion has enlarged her fair and peaceful borders in the land of the heathen, and attained a a purer and more elevated attitude in Christian countries. While the spirit of Liberty has been aroused in the slumbering breasts of thousands of people in lands of oppression and moral ignorance, and who, throwing off the shackles riveted upon them by centuries of political and religious subversion, have now taken moral and national freedom for the Lares and Penates of their hearths and homes.

In our own beautiful land, much of the political darkness that has obscured our sunny skies has been dispelled by the efforts of a people "determined to be free," and the hope may now be cherished that, before the dawn of another new year, Truth, Justice, Wisdom, and a pure Patriotism, may again rule in the councils of the Nation, and the flag of our country regain our alienated affections and obedience, by proudly floating again over a truly free and happy people.

Then, putting behind us all the irrevocable Past, let us rejoice in the gracious sunlight of the present time; let us enlarge our affections to the broad and genial philanthropy that will take into its fraternal embrace all the great brotherhood of man, and with a grateful heart still evoke the leadership of the never-erring Heavenly Guide, while we hopefully and joyfully exclaim—

> "Ring out old shapes of foul disease—
> Ring out the narrowing lust of gold—
> Ring out the thousand wars of old—
> Ring in the thousand years of peace."

